# Simple Method to Prevent Retention of Skin Staples

**Published:** 2015-01-14

**Authors:** Ken Matsuda, Minoru Shibata, Ko Hosokawa

**Affiliations:** ^a^Department of Plastic and Reconstructive Surgery, Niigata University Graduate School of Medicine, Niigata, Japan; ^b^Department of Plastic and Reconstructive Surgery, Osaka University Graduate School of Medicine, Osaka, Japan

Dear Sir,

Skin stapling devices are widely used to secure skin graft because they are easy to use and useful—especially for large grafting areas. They can save operating time and improve graft taking.^1^ However, if not removed at the appropriate time, stainless steel staples are easily buried in overgrown granulation tissue or epithelium, after which they are sometimes difficult to retrieve. Although buried staples do not generally result in adverse effects, they can provoke symptoms^2^ and result in medicolegal difficulties, as they are easily noticed in radiographs^3^ during long-term follow-up. Biodegradable skin staples have been developed to address these concerns^4^; however, the cost and inelasticity of these staples limit their utility.^5^ Although solutions for dealing with retained metal staples have been reported,^3^^,^^5^ prevention is clearly the better alternative. Skin stapling over a nonadhesive dressing material can prevent growth of tissue over staples but may result in difficulties with changing dressings and graft inspection.^3^ To prevent unintentional retention of staples, we staple skin grafts with 2-0 or 1-0 silk thread (Fig 1). These stapled silk threads do not interfere with postoperative dressing change or graft inspection. All staples are removed by lifting the silk thread at 1 week postoperatively (Fig 2). This method was successfully used for seven patients who underwent mesh skin grafting; no staples were retained. This simple and inexpensive method avoids the physical and medicolegal problems associated with staple retention.

## Figures and Tables

**Figure 1:**
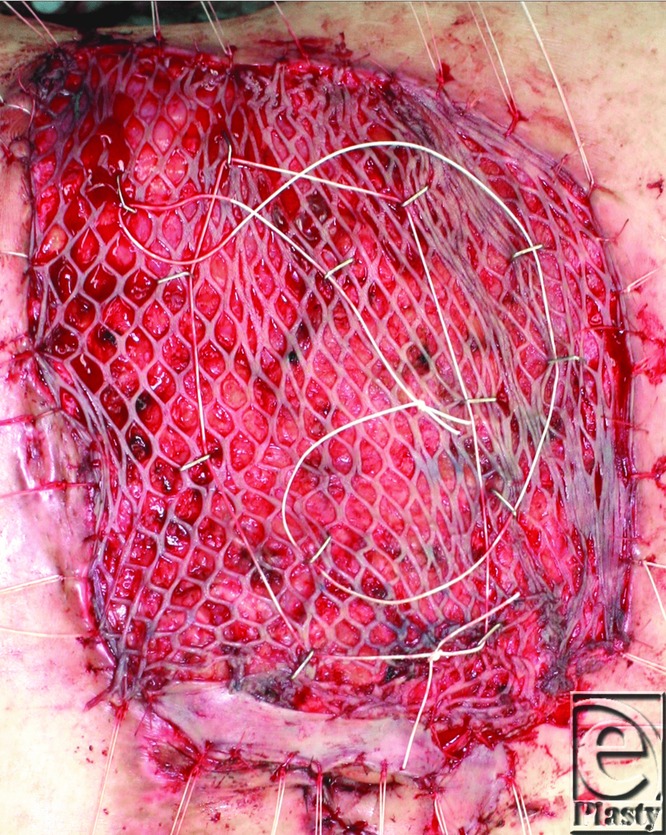
The meshed skin graft was stapled with 2-0 or 1-0 silk thread. The ends of the silk threads were tied together to avoid accidental removal.

**Figure 2:**
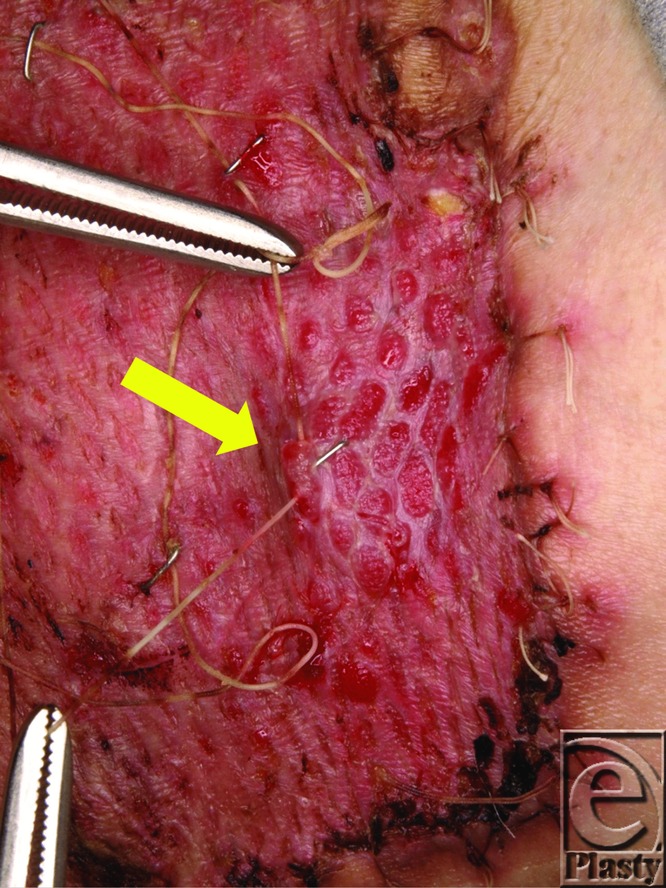
On postoperative day 7, all staples were removed by lifting the silk threads. Even on postoperative day 7, some staples were going to be buried in granulation tissue (arrow) but were easily detected because of the silk thread stapled to them.
